# Dosage Transmission Disequilibrium Test (dTDT) for Linkage and Association Detection

**DOI:** 10.1371/journal.pone.0063526

**Published:** 2013-05-14

**Authors:** Zhehao Zhang, Jen-Chyong Wang, William Howells, Peng Lin, Arpana Agrawal, Howard J. Edenberg, Jay A. Tischfield, Marc A. Schuckit, Laura J. Bierut, Alison Goate, John P. Rice

**Affiliations:** 1 Washington University School of Medicine, Department of Psychiatry, St. Louis, Missouri, United States of America; 2 Department of Biochemistry and Molecular Biology, Indiana University School of Medicine, Indianapolis, Indiana, United States of America; 3 LSB 136, Rutgers University, Piscataway, New Jersey, United States of America; 4 Department of Psychiatry, University of California San Diego, La Jolla, California, United States of America; University of Hong Kong, Hong Kong

## Abstract

Both linkage and association studies have been successfully applied to identify disease susceptibility genes with genetic markers such as microsatellites and Single Nucleotide Polymorphisms (SNPs). As one of the traditional family-based studies, the Transmission/Disequilibrium Test (TDT) measures the over-transmission of an allele in a trio from its heterozygous parents to the affected offspring and can be potentially useful to identify genetic determinants for complex disorders. However, there is reduced information when complete trio information is unavailable. In this study, we developed a novel approach to “infer” the transmission of SNPs by combining both the linkage and association data, which uses microsatellite markers from families informative for linkage together with SNP markers from the offspring who are genotyped for both linkage and a Genome-Wide Association Study (GWAS). We generalized the traditional TDT to process these inferred dosage probabilities, which we name as the dosage-TDT (dTDT). For evaluation purpose, we developed a simulation procedure to assess its operating characteristics. We applied the dTDT to the simulated data and documented the power of the dTDT under a number of different realistic scenarios. Finally, we applied our methods to a family study of alcohol dependence (COGA) and performed individual genotyping on complete families for the top signals. One SNP (rs4903712 on chromosome 14) remained significant after correcting for multiple testing Methods developed in this study can be adapted to other platforms and will have widespread applicability in genomic research when case-control GWAS data are collected in families with existing linkage data.

## Introduction

Linkage studies have been successfully used to identify many disease genes such as hyper-cholesterolaemia [1]–[3], Huntington’s disease [4] and cystic fibrosis [5]. Linkage studies allow direct observation of recombination events in a family pedigree with a limited number of generations, as well as simultaneous analysis of multiple genetic markers. The LOD score (logarithm of odds), developed by Newton E. Morton [6], is a statistical test often used for linkage analysis in human. The LOD score compares the likelihood of obtaining the test data if the two loci are indeed linked, to the likelihood of observing the same data by chance. However, this setup requires tailor-made likelihood statistics. When it comes to a multi-loci model, the situation can be even more cumbersome [7]. On the other hand, because of the requirement of a large number of families with several affected generations, linkage analysis can be less helpful when dealing with diseases of late-onset with a high mortality. Alternatively, association studies are used to identify disease susceptibility genes by comparing genetic variants between individuals with and without the disease of interest. High-throughput genotyping has allowed large-scale association studies over the entire human genome. In 2005, the first Genome-Wide Association Study (GWAS) was successfully applied on human age-related macular degeneration [8]. Since then, GWAS has been widely used to identify the association between genetic variants, typically single-nucleotide polymorphisms (SNPs), and heritable traits or diseases.

In general, there are two major types of designs that are commonly used in association research: population-based and family-based studies. As the most common population-based approach, the case-control setup compares an unrelated healthy control group and affected case group. The genotyped SNPs are investigated to identify the allele frequency differences between these two groups. The study then determines whether the SNPs are associated with the genetic trait or disease based on the statistical significance of the differences. The independent samples are typically easier to obtain in a case-control study than family samples. However, many case-control samples select independent cases from existing family data that were originally used in linkage analysis. Because cases can be over-sampled from groups with higher disease prevalence, the differences of allele frequencies in an admixture of ethnic groups may produce spurious associations. Therefore, although case-control studies have shown advantages in identifying association between the disease susceptibility and markers in a candidate gene, the results may reflect type I errors (false-positive) due to unaccounted confounding factors [9]–[11] such as population stratification [12]–[16].

Unlike the population-based studies, family-based studies are resistant to type I errors arising from population stratification. The family-based Transmission/Disequilibrium Test (TDT) measures the over-transmission of an allele from heterozygous parents to their affected offspring, in which the non-transmitted parental alleles serve, in effect, as a control group. Therefore the TDT is a robust test of association in the presence of geographical or ethnical impact from the population [17]. In the original TDT [18], a parent-proband trio is considered as a basic unit, in which a proband is the first affected family member who seeks medical attention for a genetic disorder. Assuming complete genotype information for a two allele marker locus in each trio, the TDT compares the number of heterozygous parents who transmit either allele to the affected offspring. The TDT can be constructed through a 2 by 2 table (**Table 1**). Under the null hypothesis of no associaton, the proportions 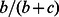 & 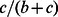
are tested against (0.5, 0.5) using a binomial (asymptotically chi-square) test with one degree of freedom:
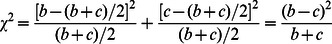
(1)


**Table 1:** Summary of the original TDT design in a 2×2 table.

	Non-transmitted allele		
Transmitted allele	M_1_	M_2_	Total
**M_1_**	*a*	*b*	*a+b*
**M_2_**	*c*	*d*	*c+d*
**Total**	*a*+*c*	*b+d*	*2n*

The letters (*a*, *b*, *c*, *d*) represent the counts of over-transmissions of an allele from the parents to affected offsprings. The number *n* denotes total number of affected offsprings and 2*n* represents the total number of parents.

Because neither genotypes nor allele frequencies are required, the TDT is considered robust to the population stratification as mentioned above.

A variety of TDT-like tests have been suggested starting with Rubinstein et al [19]. Curtis and Sham studied a multi-allelic TDT with incorporation of missing parents [20], [21]. This was extended by Spielman et al and Horvath et al [22], [23] with the TDT applied to different family structures in their sib-ship tests. For an allele of interest at a marker locus, the sib-ship test essentially compares the frequency of that allele among affected individuals with the frequency of the allele among unaffected individuals, which allows the TDT to be applied to diseases with late age of onset, such as non-insulin-dependent diabetes, cardiovascular diseases, Alzheimer’s disease, and other diseases related to aging. Several studies also discussed the application of the TDT for mapping quantitative trait loci [24]–[30]. Gordon et al.’s TDTae allows for genotyping errors in the analysis and accommodates various error models [31]. As discussed above, multiple affected and unaffected siblings are often collected and used for both linkage and association analysis. The family-based association test (FBAT) generalized the TDT model on various phenotypic traits and multiple markers [32]–[36]. Instead of using data from only the heterozygous parents as in the TDT, the affected-family based controls (AFBAC) method [37] is developed to take advantage of all the parental information. But the trade-off of this setup is its vulnerability to population stratification as genotype frequencies are not irrelevant in this test [37], [38]. Another extension of the TDT, the pedigree disequilibrium test (PDT), is specifically designed for analyzing the Linkage Disequilibrium (LD, the non-random association of alleles at two or more loci) in general pedigrees, which has been successfully applied on a number of complex traits such as diabetes [38], [39]. Further, as a more powerful development to the PDT, the presence of linkage (APL) is used to handle diseases of late-onset [38], [40]. However, in spite of the divergences as well as the great promise of these TDT-type analyses [9], [24], [41], one primary limitation that most of these extensions encounter is the dependence on completeness of the genotype information for all trio members in a single test and lack of scalability on utilizing both the linkage and association data in a study.

Disorders can often have genotype information from only one parent of the affected individuals. As a common practice, these trios are simply discarded [42] though this can result in considerable loss of information and bias to the association study [20], [38]. Several studies have been proposed to allow TDT to handle missing parental genotypic information [20], [22], [43]–[50]. Within these studies, the missing parental genotypes are mostly reconstructed based on the assumption that they are missing completely at random and do not depend on the genotypes themselves [51]. However, this assumption may not hold true and the probability that a genotype is missing may rely on the unobserved alleles [38], [52], [53]. Furthermore, these approaches are not designed for pedigrees with missing genotypes on the proband when both linkage and association data are available.

Against this background, we note this is a two step procedure. In the first step, the SNP data is used with both parents missing, so that the analysis depends on external allele frequency estimates and is sensitive to population stratification. In the second step, individual genotyping is performed on the parents for the top SNPs from step one, so this step is a traditional TDT and insensitive to the potential biases in step one. Accordingly, having available family DNA is needed to avoid false positive results.

## Methods

As stated above, the traditional TDT requires complete genotypic information from all members of the nuclear families. However, obtaining all genotypes cannot always be feasible for some diseases or families. Therefore the traditional TDT-type studies may not be useful to identify the presence of genetic determinants in data with relatively small amounts of complete trio information. One way to solve this type of issue is to reconstruct the missing parental genotypes under the assumption that they follow the probability distribution of the fully observed cases. However, most studies designed for this purpose do not incorporate the impact from LD and thus may introduce bias to the results. On the other hand, there are data available that have genotype information on both microsatellite and SNP markers for diseases; one example is alcoholism [54]. With both the linkage and association data, usually the microsatellite genotypic information from families for linkage analysis together with SNP data from the offspring who are genotyped for both linkage and GWAS, we can “infer” the transmission of SNPs for the rest of the family members who have not been genotyped on SNPs. We call these family members the “missing individuals”. In detail, first we generate the combined pedigrees in which each individual has both the linkage and GWAS genotype data filled in. Genotypes that those individuals do not have will be taken as missing data in the combined pedigrees. Then we use the program MERLIN [55] to read these combined pedigrees as input and infer the dosage probabilities of dense SNP genotypes for these missing individuals (see section *Genotype Inference of Familial Individuals* for more details). All the trio combinations from the inferred pedigrees are extracted on the condition that the children were affected and at least one parent in the trio was genotyped on microsatellite markers. The dosage-TDT that we have developed in this study is applied on these trio pedigrees using their inferred dosage probabilities. By incorporating the family linkage information into the GWAS data, we can potentially have higher power to detect association between our genotypic markers and the disease susceptibility alleles.

### Dosage Transmission Disequilibrium Test (dTDT)

#### Common map of both linkage & GWAS data

The common map of both linkage and GWAS data is designed in the following way. With the genetic position of the linkage markers (microsatellite as in here) and physical position of both linkage and GWAS markers (microsatellite and SNPs), the genetic positions of all the GWAS markers (SNPs) are calculated based on equation (2):
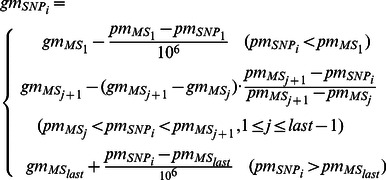
(2)where *gm* denotes the genetic position of a marker, in *centi-Morgan* (*cM*) unit; *pm* denotes the physical position of a marker, in *base pair* units; *MS_last_* is the last microsatellite marker on a chromosome, 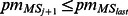. Because at both ends of a chromosome when the physical position of a SNP is either smaller than the 1^st^ microsatellite marker or larger than the last microsatellite marker, there is only one microsatellite marker that can be referred to compute the genetic position for the SNP. We simply use the convention that 1*cM* ≈ 10^6^ base pairs to convert a SNP’s physical map to its genetic map. While a SNP is in between two microsatellite markers, we use the ratio 
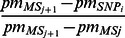
 and multiply this ratio with the genetic distance between these two microsatellite markers 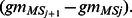 In this way we compute the *relative* genetic position of a SNP marker to the microsatellite marker that’s next to it.

#### Genotype inference of familial individuals

**Figure 1:**
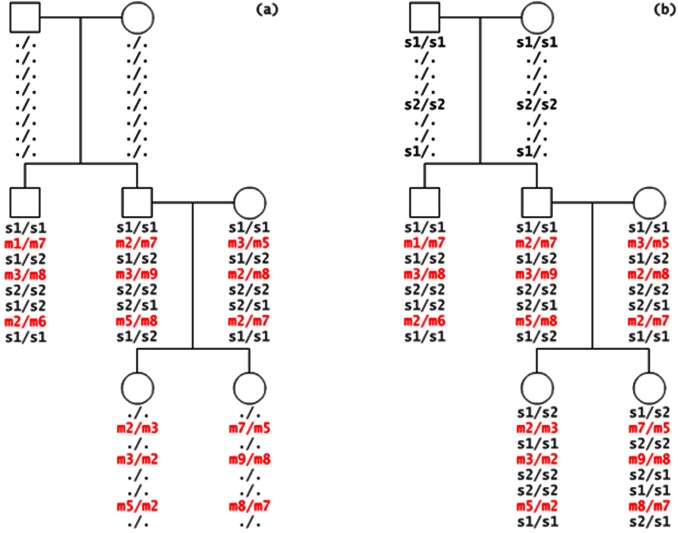
Demonstration of genotype inference within a family. (a) The observed data, which consist of genotypes at a series of microsatellite and SNP markers. A subset of microsatellite markers has been typed in all individuals except for founders (red), whereas both microsatellite and SNP markers have been typed in only a few selected common individuals (black). (b) Genotypes of dense SNPs for missing individuals are inferred by comparing the haplotypes they share with the common individuals.

Initially, many approaches implicitly imputed missing genotypes based on the potential genotype distribution in a family [56]–[58]. In practice, the genetic linkage implied that family members share a certain degree of similarity through their “identical-by-descent” (IBD) regions on the chromosomes. In this way, genotypes of the non-typed markers for these family members can be inferred according to their shared IBD with the other relatives. **Figure 1** illustrates the procedure of this genotype inference. As shown in the figure, a subset of microsatellite markers has been typed for all the family members except the founders (red), whereas both microsatellite and SNP markers have been typed in only a few selected common individuals (black). Genotypes of the dense SNPs for missing individuals can be inferred by comparing the haplotypes that are IBD with the other individuals in the family. Several studies have been published on the genotype imputation procedures described above [59], [60]. These procedures are implemented in programs such as MERLIN [55], [61] and MENDEL [62], [63], using one of the pedigree analysis algorithms such as the Lander-Green [64] or Elston-Stewart [65] algorithms, or Monte Carlo sampling [66], [67]. Merlin uses sparse trees to represent gene flow in pedigrees and is considered as one of the fastest packages among packages implementing the same algorithms such as Allegro [68] and Genehunter [69]. In this study, we use MERLIN to infer the dosage probabilities. The output of this program includes the most likely genotypes, the expected number of copies for the tested alleles (0, 1, or 2 with genotype observed), and the posterior probabilities (dosage probabilities) of the three alternative genotypes [70]. Because a large number of related individuals are included, this family-based genotype inference is expected to improve the power of association tests [59]. Furthermore, when a GWA scan follows a linkage study, only a proportion of individuals may need to be genotyped and the inferred genotypes can be useful for the next step in the association analysis.

#### Dosage-TDT

Because the traditional TDT is a simple representation of the *χ* ^2^ statistics, it requires single counts of the transmitted/non-transmitted alleles from the heterozygous parents to the affected offspring. Thus the inferred dosage probabilities cannot be processed through this setup. In this study, we generalize the original TDT by taking all possible allele transmissions in a pedigree into account. **Table 2** shows the dosage probabilities of three alternative genotypes (1/1, 1/2 and 2/2) in a trio (named a *trio-dosage set* in this work) from the inferred results. **Table 3** lists all 11 TDT-informative allele transmissions in a trio where at least one of the parents is heterozygous. The values of *b_i_* and *c_i_* used in the *χ* ^2^ calculation of the TDT in each trio *i* are calculated by summing up the probabilities across all these 11 types of transmissions. Let *t* denote the probability that allele 1 is transmitted by a heterozygote parent of an affected child. We can then write the dosage probabilities of a child in terms of the dosage probabilities of its parents and *t* as follows:
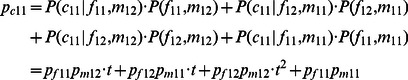
(3)

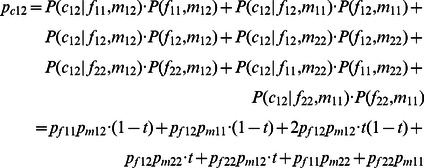
(4)

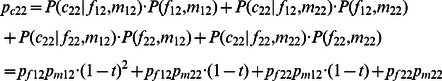
(5)


**Table 2:** Dosage probabilities in a trio (denoted as a trio-dosage set).

	Genotype		
	1/1	1/2	2/2
Father	*p_f11_*	*p_f12_*	*p_f22_*
Mother	*p_m11_*	*p_m12_*	*p_m22_*
Child	*p_c11_*	*p_c12_*	*p_c22_*

**Table 3:** Calculation of *b_i_* and *c_i_* in terms of dosage probabilities and *t* for the *i* ^th^ trio with all 11 TDT-informative transmissions.

	1/1–1/2|1/1	1/1–1/2|1/2	1/2–1/1|1/1	1/2–1/1|1/2	1/2–1/2|1/1	1/2–1/2|1/2	1/2–1/2|2/2	1/2–2/2|1/2	1/2–2/2|2/2	2/2–1/2|1/2	2/2–1/2|2/2	Sum
b_i_	*p_f11_p_m12_•t*		*p_f12_p_m11_•t*		*2p_f12_p_m12_• t^2^*	*2p_f12_p_m12_• t(1-t)*		*p_f12_p_m22_•t*		*p_f22_p_m12_•t*		*(p_f12_+p_m12_) •t*
c_i_		*p_f11_p_m12_• (1-t)*		*p_f12_p_m11_• (1-t)*		*2p_f12_p_m12_• (1-t)*	*2p_f12_p_m12_• (1-t)^2^*		*p_f12_p_m22_• (1-t)*		*p_f22_p_m12_• (1-t)*	*(p_f12_+p_m12_) • (1-t)*

*t* denotes the possibility that allele 1 is transmitted by a heterozygote; and (*1-t*) is the possibility that allele 2 is transmitted.

Thus, the frequencies of allele 1 and 2 of a child appearing in a trio are as follows:
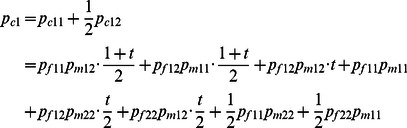
(6)

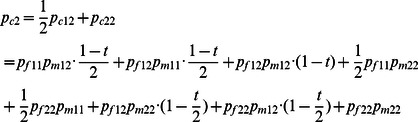
(7)

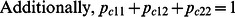
(8)


Based on equations (3) to (8), we can derive that:
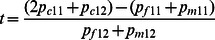
(9)


In summary, the sum of all 11 informative transmissions for *b_i_* and *c_i_* can be written as:
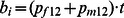
(10)

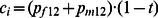
(11)where *i* represents the *i* ^th^ trio. By substituting *t* from equation (9) into (10) and (11) and summing up *b_i_* and *c_i_* for all trios, we can compute the *χ* ^2^ values for each SNP:
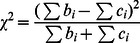
(12)which follows a one degree freedom χ2 distribution under the null hypothesis of no association. We name this generalized TDT the dosage TDT (dTDT). The original TDT proposed by Spielman et al [18] can then be considered as a special case when t and the dosage probabilities in a trio-dosage set is 0 or 1.

The beauty of the above equations is the denominator from *t* can be canceled out with the one in *b_i_* and *c_i_* thus equation (12) can be further written as:
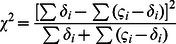
(13)in which we denote



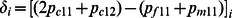
(14)

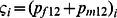
(15)for each trio *i*. Using form (13) can be computationally efficient.

### Simulation

The dTDT makes it possible to process the inferred dosage probabilities of the un-genotyped SNPs for those missing individuals in a nuclear family. As a follow-up study of this generalized TDT approach, we develop a simulation to investigate how the power changes for association detection with different inputs. In this simulation, we generate multiple sets of trios under various settings. Each set has 1,000 trios. Each trio has one affected child. Because we focus on the interaction between SNP and microsatellite markers, only one SNP marker and one microsatellite marker are simulated. In each trio, the microsatellite markers are assigned to both the parents and the child. SNPs are only assigned to the child. The parents who do not have such SNP markers are considered as the missing individuals and their SNP genotypes are inferred by MERLIN. The dTDT is then used to process the inferred dosage probabilities and p-values are reported from the *χ* ^2^ statistics.

#### Generating SNPs

Denote the low and high risk alleles at a disease locus *D* as *D_1_* and *D_2_*, with population frequencies *p_1_* and *p_2_*. Assuming Hardy-Weinberg equilibrium, the population prevalence (*K*) of the disease is
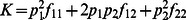
(16)where *f_11_*, *f_12_* and *f_22_* are the penetrances of the three genotypes *D_1_D_1_*, *D_1_D_2_* and *D_2_D_2_*.

We have considered three disease models: dominant, recessive and co-dominant. The combinations of the penetrances in these three models are designed as follows: dominant (*f*_11_<*f*_12_ = *f*_22_), recessive (*f*_11_ = *f*_12_<*f*_22_) and co-dominant (*f*_11_<*f*_12_ = ½ *f*_22_).

With *K* and *f* predefined, we can compute *p*_1_ and *p*_2_ using the following equations:
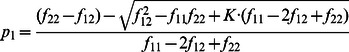
(17)

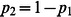
(18)


Denote the haplotype frequencies of disease locus *D* and SNP locus *S* as *h_11_*, *h_12_*, *h_21_* and *h_22_*. On condition of the child being affected, the probabilities of different haplotypes in a child can be calculated through:
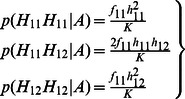


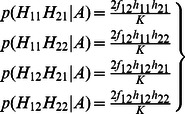
(19)

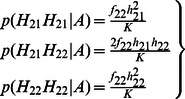



With a predefined correlation coefficient (*R*) of linkage disequilibrium (LD) between D and S, we can derive the haplotype frequencies as follows:
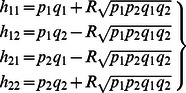
(20)where *q_1_* and *q_2_* are the population frequencies of the SNP alleles *S_1_* and *S_2_*. To simplify our model, we will assume *p_i_* = *q_i_*, where *i* = 1 or 2. The rationale behind this is that if the SNP marker and disease allele have very different frequencies, then *R* ^2^ is small and there is little power. Keeping both frequencies equal allow *R* to vary the full range from −1 to +1.

By substituting (20) into (19), we can assign the genotypes (*S_1_S_1_*, *S_1_S_2_*, or *S_2_S_2_*) at locus *S* to the affected children based on these derived frequencies:
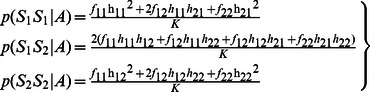
(21)


#### Generating microsatellites

Denote *M_i_* as the microsatellite marker from the parents. Because of a large number of polymorphisms (alleles) for a microsatellite marker, we assume that our microsatellite marker is completely informative (i.e., each parent is heterozygous at the microsatellite locus M) and assign alleles *M_1_* & *M_2_* to the father and *M_3_* & *M_4_* to the mother. Then we randomly select *M_1_* & *M_3_*, *M_2_* & *M_4_*, *M_1_* & *M_4_* or *M_2_* & *M_3_* equally with 0.25 probabilities as the microsatellite genotype for the child.

#### Parameter settings

Without losing biological meaning, i.e. with valid *p_i_* ∈(0, 1.0] (*i* = 1 or 2 and *p*_1_+ *p*_2_ = 1), but also with a good coverage of possible natural phenomena, we predefine the following values for the parameters to generate each set of trios:

*N*: the number of trios = 1,000;

*K*: prevalence = 0.01, 0.1, or 0.2;

*R*: correlation coefficient of LD between *D* and *S* = 0.5, 0.7, 0.9, or 1.0 (as negative value of *R* does not produce informative divergence from the result using positive value of *R*, we are only considering positive value of *R* herewith);

*f* or *g*: penetrance of disease genotype *D_i_D_i_* or *D_i_D_j_*, where *i* or *j* = 1 or 2 and *i* ≠ *j*. To simplify the notation, here we use *f* to denote *f*_11_ and *g* to denote *f*_12_ or *f*_22_. As noted, we separate the disease models as dominant (*f*, *g*, *g*), recessive (*f*, *f*, *g*), and co-dominant (*f*, 0.5*g*, *g*) where *f* = 0.0, 0.1*K*, 0.3*K*, 0.5*K*, 0.7*K*, or 0.9*K* and *g* = 1.1*K*, 0.5, 0.7, 0.9, or 1.0.

These values are first permuted to generate all their possible combinations then any combination that produces invalid *p*, i.e.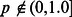, is excluded. Under each setting, we produce 1,000 trios based on equation (15) for SNPs in the affected offsprings. Parental SNPs are inferred by MERLIN and dTDT is used to process the inferred dosage probabilities.

### Application to Alcohol Dependence

Alcohol dependence is a serious psychiatric disorder in which an individual is characterized as having harmful consequences of repeated or compulsive alcohol use, and (sometimes) physiological dependence on alcohol (i.e., tolerance and/or symptoms of withdrawal) [71], [72]. During 2001–2005, excessive alcohol use contributed to about 79,000 deaths and 2.3 million years of potential life lost in the United States [73]. Excessive alcohol consumption, the third leading cause of preventable death in the United States, can cause damage to the central and peripheral nervous system, and to nearly every organ system in the body [74], [75]. It is reported that alcohol dependence affects about 12% of American adults across their lifetime [76]. As a complex disease, alcohol dependence can be influenced by various factors such as genetic susceptibility, environmental influences and interactions among genes or between genes and environment.

The nine-site national Collaborative study On the Genetics of Alcoholism (COGA) funded by National Institute of Alcohol and Alcoholism (NIAAA) aims to identify and characterize genes that affect the susceptibility to develop alcohol dependence and related phenotypes. COGA is applying multiple strategies for genetic research. The most densely affected, multiplex alcoholic families were used in a multi-wave family-based linkage study. 2,283 out of 2,459 individuals from 262 families were genotyped using microsatellite markers in Wave I and Wave II (data denoted as Map03MS) (**Table 4**) [54]. At Wave III, another 1,442 out of 2,106 individuals from 312 families were selected for microsatellite genotyping by the Mammalian Genotyping Service (MGS) from Marshfield Clinic (data denoted as MarshfieldMS) (**Table 4**). Combined data from all three waves are denoted as LinkageMS in this study. COGA also has high throughput GWAS data with over 1 million SNP markers from 1,884 independent individuals, generated by the Center for Inherited Disease Research (CIDR) (data denoted as CIDRSNP) (Edenberg et al., 2010). The GWAS data include 566 mutual individuals chosen from the LinkageMS families.

**Table 4:** Number of total and genotyped individuals, and corresponding number of families that these individuals are selected from.

	Total Individuals(*ind_total_*)	Number of Genotyped Individuals on microsatellite markers (*ind_MS_*)	Number of Families
Map03_MS_ EA	2,037	1,926 (94.55%)	219
Map03_MS_ AA	335	283 (84.48%)	35
Map03_MS_ Mixed	87	74 (85.06%)	8
Marshfield_MS_ EA	1,530	1,090 (71.24%)	234
Marshfield_MS_ AA	570	347 (60.88%)	77
Marshfield_MS_ Mixed	6	5 (83.33%)	1

All participants agreed to share their DNA and phenotypic information for research purposes and provided written informed consent following instructions from institutional review boards at all data collection sites. The study was approved by the institutional review board at each COGA site, with the OHRP Assurance Numbers being**:** FWA00003624 (SUNY Research Foundation), FWA00007125 (University of Connecticut), FWA00003544 (Indiana University), FWA00003007 (University of Iowa), FWA00004069 (Veterans Medical Research Foundation/UCSD), FWA00002284 (Washington University), FWA00003518 (Southwest Foundation for Biomedical Research), FWA00003913 (Rutgers, The State University of New Jersey) and FWA00005287 (Virginia Commonwealth University).

As described above, the dTDT uses the inferred dosage probabilities of dense SNPs for association detection. The COGA family data provides us such an opportunity to integrate the information from both linkage and association studies.

**Figure 2:**
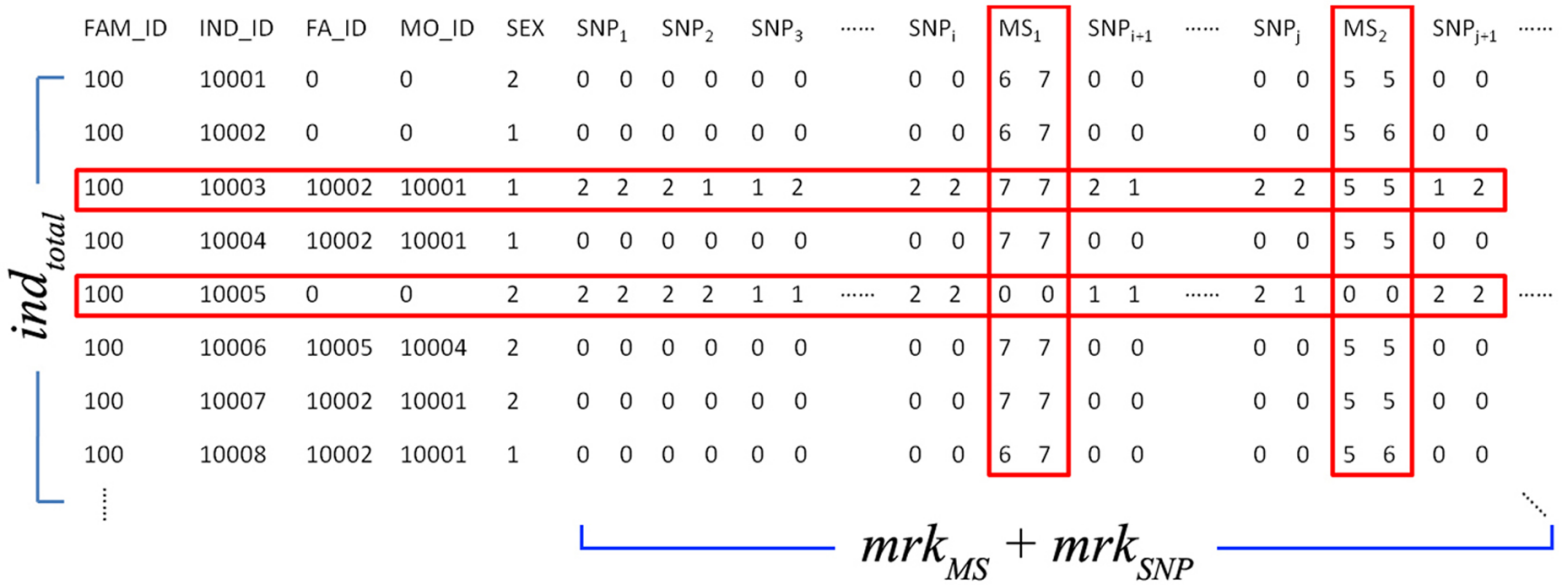
Combined pedigree structure used in inference. The pedigree has *ind_total_* individuals and (*mrkMS*+*mrk_SNP_*) markers. Most individuals have been genotyped on microsatellite markers. *ind_common_* out of *ind_total_* individuals are selected for SNP genotyping. Microsatellite and SNP markers are mapped based on their genetic positions. Missing SNPs of (*ind_total_* - *ind_common_*) will be inferred by MERLIN –infer.

In this study, we first generate the combined pedigrees with both the LinkageMS and CIDRSNP genotype data from COGA. **Figure 2** demonstrates the structure of the combined pedigrees. Most individuals in the combined pedigrees were genotyped on microsatellite markers. A subset of individuals in the pedigrees was genotyped for dense SNPs. These individuals include one affected child in each of the families and other unrelated members chosen as a control group. All other individuals who have not been genotyped on SNPs are considered as the missing individuals. We use the program MERLIN [55] to read these combined pedigrees as input and infer the dosage probabilities of dense SNP genotypes for these missing individuals (described in detail below). All the trio combinations from the inferred pedigrees are extracted on the condition that the children were affected and at least one parent in the trio was genotyped on microsatellite markers. The dTDT is applied on these trio pedigrees using their inferred dosage probabilities. In addition, PLINK ^77^ is used to conduct a standard case-control study on the CIDRSNP data. With the idea that making use of all the available sample data would increase the power for association detection, we further combine the results from both dTDT and case-control study through the MH test [78].

#### Data sets

The Map03MS data have 219 European American (EA), 35 African American (AA) and eight mixed families. 2,283 individuals from these 262 families were genotyped on 328 microsatellite markers. The MarshfieldMS data contain 234 EA, 77 AA and one mixed families, with a total of 1,442 individuals genotyped on 394 microsatellite markers. 1,041,304 SNPs were genotyped for 1,399 EA and 485 AA individuals in the CIDRSNP GWAS. (**Tables 4** **,** **5** **and** **6**).

**Table 5:** Number of common individuals in EA group genotyped on both microsatellite & SNP markers, and corresponding number of families.

	Number of Common Individuals (*ind_common_*)	Number of Families with Common Individuals	Number of Families with *m* Common Individuals				
			*m = *	1	2	3	4
Map03_MS_ × CIDR_SNP_ EA	260	208		167	32	7	2
Marshfield_MS_ × CIDR_SNP_ EA	211	190		169	21	-	-

In total, 471 (13.2%) out of 3,567 individuals were selected for SNP validation genotyping.

**Table 6:** Summary of microsatellite and SNP markers in EA group before and after cleaning.

	Total Number of Raw microsatellite markers	Number of microsatellite markers after cleaning (*mrk_MS_*)	Number of CIDR SNPs after cleaning (*mrk_SNP_*)*
Map03_MS_ EA	328	290	801,273
Marshfield_MS_ EA	394	336	801,286

* compared to 1,041,304 SNPs before cleaning.

**Figure 3:**
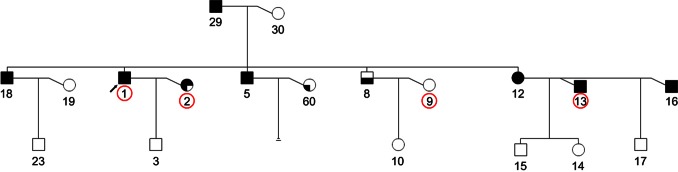
Pedigree of one Map03 family (FAM_ID 20059). Common individuals (#1, 2, 9 and 13, from left to right) are genotyped on both microsatellite and SNP markers (circled in red). Box shadowed in upper left: AB, alcohol abuse; shadowed in upper left & right: AD, alcohol dependence (DSM III-R Diagnosis). Box shadowed in lower left: PROB, probable; shadowed in lower left & right: DEF, definite (Feighner Diagnosis).

With AA and mixed families excluded, we have 3,016 out of 3,567 EA individuals from 453 families genotyped on microsatellite markers in the LinkageMS data. 471 of these individuals in 398 linkage families were selected for SNP genotyping (known as the mutual individuals) (**Table 6**), including 41 individuals without microsatellite genotyping data. For GWAS, from each of these 398 families, one affected child (normally the proband) was selected as the case and other biologically unrelated family member(s) were used as the control. In total, 1,399 EA CIDRSNP individuals consist of 847 cases and 552 controls. **Figure 3** shows the pedigree of one of the LinkageMS EA families (FAM_ID 20059). This family has four mutual individuals. Except for proband #1, all the other three (#2, 9, and 13) selected for GWAS are relatives by affinity to this family.

#### Marker cleaning & mapping

**Figure 4:**
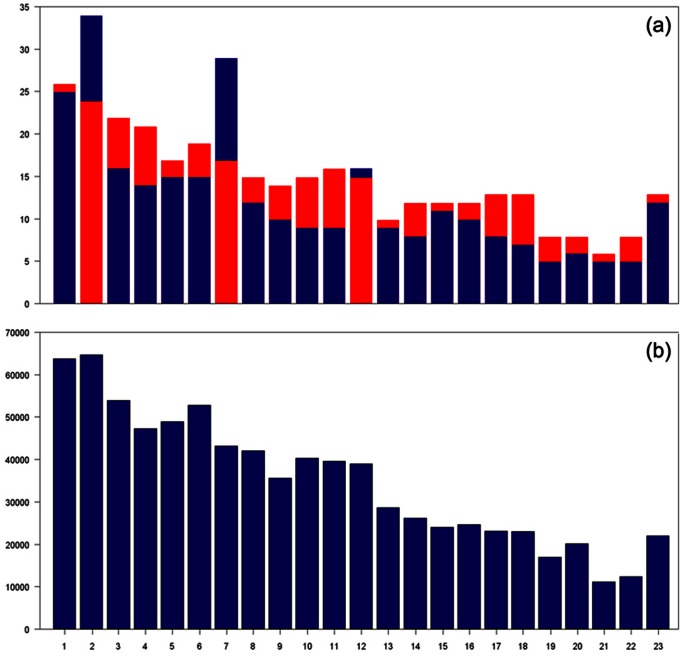
Distribution of cleaned microsatellite and SNP markers on 23 chromosomes in EA families. These markers are used in the combined pedigrees for genotype inference. (a) distribution of microsatellite markers on 23 chromosomes in Map03 (blue) and Marshfield (red) (overlapped); (b) distribution of SNPs on 23 chromosomes in Map03 and Marshfield. Because the difference of SNPs numbers in these two datasets is trivial, we only display the distribution of SNPs in Map03.

In order to match up the genetic positions of all microsatellite and SNP markers, 38 microsatellite markers in Map03 and 58 microsatellite markers in Marshfield were excluded because of their missing physical positions. ∼200,000 SNPs with low minor allele frequency (≤5%) were excluded. In consideration of any possible impact from linkage disequilibrium (LD), we exclude ∼1,500 SNPs that are within 1,000 base pairs flanking each microsatellite marker. We use equation (2) to create the common map for these microsatellite and SNP markers. A comparison of the numbers of microsatellite and SNP markers before and after cleaning is given in **Table 6**. **Figure 4** shows the distribution of these cleaned markers in EA families. With these cleaned and mapped markers, we create new pedigrees with the Linkage_MS_ and CIDR_SNP_ data combined together. One combined pedigree has *ind_total_* individuals (in rows) with (*mrk_MS_*+*mrk_SNP_*) markers (in columns). Missing SNPs of (*ind_total_* - *ind_common_*) individuals were inferred by MERLIN. (**Figure 1**).

#### dTDT and mantel-Haenszel test

In this study, we apply both the dTDT and Mantel-Haenszel (MH) tests to the COGA data. The MH test was first proposed by Mantel and Haenszel in 1959 [78]. The method has been widely applied to analysis of contingency tables (normally 2 × 2) and comparison of results from different treatments. In case-control studies, a 2 × 2 table is typically used. The discrepancy between observed and expected values in each cell from the table is evaluated by *χ* ^2^ test with one degree of freedom. Comparatively, because the dTDT only takes account of values of *b* and *c*, the test can be constructed by a 1 × 2 table instead. To maximally benefit from all sample data and multiple studies, we extend the MH test to pool results on each SNP from these two contingency tables in both case-control study and dTDT. Calculation of each term in the MH test is shown in **Table 7**. Having the Observed & Expected values and Variances from case-control study and dTDT, terms in MH test can be written as the sums of corresponding values these two tests. The null hypothesis assumes no association between markers and disease.

**Table 7:** Calculations of Observed & Expected values, Variance, *χ* ^2^ test in Case-Control study, dTDT and MH test.

	Case-Control study			dTDT				MH test		
Structure		Case	Control	NT						
				allele 1			allele 2	–		
	# allele 1	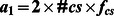	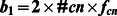	T	allele 1	–	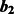			
	# allele 2	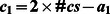	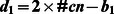		allele 2	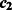	–			
Total (N)	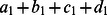			–					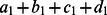	
Observed (O)	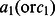			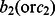					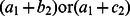 *	
Expected (E)	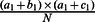			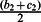					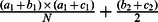	
Variance	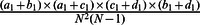			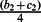					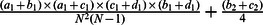	
*χ* ^2^ test	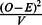			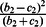					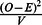	

Number of Cases (#*cs*) = 847; Number of Controls (#*cn*) = 552; *f_cs_*: allele frequency in cases; *f_cn_*: allele frequency in controls; T is short for Transmitted; NT is short for Non-Transmitted. *b_2_* = *Σb_i_* and *c_2_* = *Σc_i_*. Using either *a*_1_ or *c*_1_ in Case-Control study will give the same results.

* equivalent to 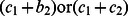.

## Results

### Simulation

**Figure 5:**
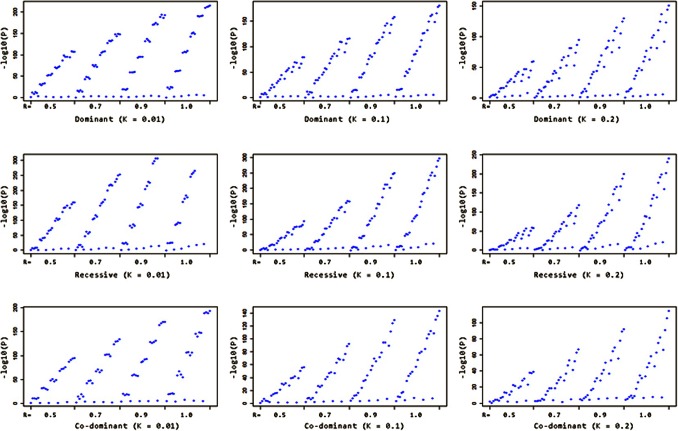
–log_10_(*P*) distribution of nine disease models from the simulation results. Graphs from the top row to the bottom row represent the dominant, recessive and codominant models respectively, and from left to right represent the results with three different *K* values (0.01, 0.1 and 0.2). Each blue dot corresponds to a –log_10_(*p-value*) under that specific setting. Every graph is broken down into four bins with R = 0.5, 0.7, 0.9 and 1.0. Within each bin, the –log_10_(*p-values*) are ordered by descending *f*_11_ and increasing *f*_12_ & *f*_22_.

We separate the simulation results into nine groups that are combinations of three disease models and three *K* values (0.01, 0.1 and 0.2). In each group there are 100∼120 settings with different *R* and *f* values. With each setting we generate 1,000 trios and replicate the inference and dTDT procedures. Because of the large number of these settings (1,320 in total), we attach the results as in supplement tables. A plot of the 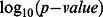
for these models is shown in **Figure 5**. In the figure, graphs from the top row to the bottom row represent the dominant, recessive and codominant models respectively, and from left to right represent the results with three different *K* values (0.01, 0.1 and 0.2). Each blue dot corresponds to a/under that specific setting.

Because there are many factors interacting with each other, we will start with a general comparison of different models then look at the impact from one or two factors while constraining the others constant.

In general, reading the values of 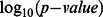
from each model, we find that a rare (*K* = 0.01) recessive disease model produces higher power compared to common (*K* = 0.1 or 0.2), co-dominant or dominant disease models. Meanwhile, high *R* value (0.9 or 1.0) also helps increase dTDT’s ability to detect signals. This is because in a rare recessive case, markers with high LD to the disease allele both parents are heterozygous and both transmit the recessive risk allele to their offspring. Our findings from the simulation validate what we already observed in the biological phenomenon.

In the figure, each graph is broken down into four bins having R = 0.5, 0.7, 0.9 and 1.0. Interestingly, within each bin, when 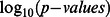 are ordered by descending *f*_11_ (from 0.9K to 0.0) and increasing *f*_12_ & *f*_22_ (from 1.1*K* to 1.0), it shows a noticeable increasing trend as shown on the graphs. Meanwhile, when *f*_22_ is small ( = 1.1*K*), the blue dots are close to the bottom line on each graph. We note that in order to have enough power to detect the signals, we need to have relatively distinguishable penetrances (i.e. *f* cannot be too close to *g*) in the model. Indeed, there is no information when all three penetrances are equal to *K*.

When we generate the trios, we use a roulette wheel algorithm to assign SNPs to the children. This randomness is reflected on the graphs as the dots spread in some irregular patterns. Reading the graphs from left to right, we can see that with low prevalence (*K* = 0.01) the dots appear in clear clusters. Each cluster corresponds to a specific *f*_11_ value. Taking the top left graph (dominant with *K* = 0.01) as an example, *f*_11_ changes in the order of 0.9*K*, 0.7*K*, 0.5*K*, 0.3*K*, 0.1*K* and 0.0. Within each cluster, *f*_12_ & *f*_22_ increase in the order of 1.1*K*, 0.5, 0.9 and 1.0. This clustering holds true in the other two disease models (recessive and co-dominant) when *K* is small (*K* = 0.01) except some *f*_11_ valued clusters are missing because combinations with invalid *p* were excluded. In summary, the above observation indicates that *f*_11_ has a higher impact to the power than *f*_12_ & *f*_22_ do in a rare disease model. As the prevalence increases (*K* = 0.1 and 0.2), the clustering effect gradually disappears. In each *R* valued bin when *K* is large (0.1 or 0.2), though the penetrances are sorted in the same order as mentioned above, the dots represent certain continuity instead of clustering. This shows that, in a common disease model, *f*_11_ is not the only or the most effective factor as it is in a rare disease model. Other factors start to interact with each other. Especially when *K* = 0.2 and *R* = 1.0 (the fourth bin in the three graphs on the right), the dots appear in clear fan-shaped sectors. This irregularity can be partially explained by the sensitivity to randomness of the model under such setting, i.e., small changes of the parameters can have high impact on the results.

We can further see how the penetrances differ by looking at the slope of the trend in each bin. Apparently as the value of *R* increases across the bins, the slope of the trend also increases. This is because when *R* is high (such as 0.9 and 1.0), the same degree of lift in the penetrances will add more power and move/more quickly to its next level compared to the situation when *R* is low (such as 0.5). From another point of view, we can imagine these slopes as the (first) derivatives of a convex function in terms of *R*. On this convex curve, as *R* moves along to its rightmost end (increases), the derivative of the function increases and the function value (power) improves faster.

### Application to Alcohol Dependence

In a recent work in a case-control study using GWAS data on the COGA sample, Edenberg et al [79] identified the most significant SNP rs10511260 on chromosome 3 with *p*-value (P) = 3.4×10*^−^* ^6^. A cluster of SNPs was found in a region of chromosome 11 with *p*-values ranging from 4.8×10*^−^* ^5^ to 6.9×10*^−^* ^4^. No single SNP showed genome-wide significance (5×10*^−^* ^8^). In the following sections, we will compare our results from dTDT on COGA data with these findings from Edenberg et al’s work.

#### dTDT on COGA data

To apply the dTDT on each SNP from COGA, we re-build the inferred pedigrees by extracting all trio combinations in which every child in a trio must be affected and at least one parent was genotyped on microsatellite markers. Because one family can have more than one affected child, the parents can be found in more than one trio. For instance, the family (FAM_ID 20059) as shown in **Figure 3** has four affected children (#18, 1, 5 and 12, from left to right). Mother (#30) was genotyped on microsatellite markers. Therefore we have four trios from this family. In total, 893 trios from 323 families with 1,654 individuals in the Linkage_MS_ EA group were extracted and used to build the trio-dosage pedigrees. 166 SNPs are found with *p*-values <10*^−^* ^4^. This is compared to 93 SNPs at the same level in Edenberg et al’s paper [79]. Several clusters of SNPs on chromosome 7, 8 and 22 have *p*-values ≤10*^−^* ^5^. However, further analysis with MH statistics does not yield consistent results with dTDT. This may be primarily due to the relatively small sample size used in dTDT and accuracy of the “–infer” program.

#### Combining case-Control study and dTDT

**Figure 6:**
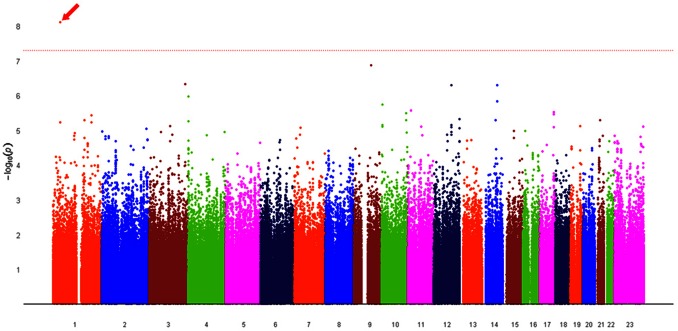
Manhattan plot of –log_10_P from MH test across all 23 chromosomes for Linkage_MS_ EA data. The dashed red line shows the genome-wide significant level –log_10_(5 × 10*^−^* ^8^) ≈ 7.3. SNP rs11583322 has given –log_10_P ≈ 8.1 above this level that lies in gene STK40.

With dTDT and case-control analysis applied to the Linkage_MS_ and CIDR_SNP_ data respectively, we compute the *p*-values of MH test based on calculations in **Table 7**. **Figure 6** shows the Manhattan plots of *–log_10_P* across all 23 chromosomes from the MH test. The most significant SNP (rs11583322) on chromosome 1 gives a *p*-value = 1.10×10*^−^* ^8^ that meets the GWAS significance level. This SNP lies in the gene Serine/Threonine Kinase 40 (*STK40*) that connects pluripotency factor Oct4 to the Erk/MAPK pathway controls extraembryonic endoderm differentiation [80]. **Table 8** lists the top SNPs with MH test *p*-values <10*^−^* ^5^ and their corresponding case-control study and dTDT *p*-values. From the table we can see that the *p*-value of each SNP in the MH test is approximately the product of *p*-values in the other two tests. However, SNPs with high rankings by *p*-values in the MH test do not systematically correspond to high rankings in the individual tests. The *p*-values in the dTDT share the highest variance (2.01×10*^−^* ^3^) among the three tests because of the randomness introduced by the inference procedure as well as the difference in sample sizes across the tests. A larger sample size will likely increase the power and generate more robust test results. In total, we have 257 SNPs in 75 genes with *p*-values <10*^−^* ^4^. 14 SNPs at the same level of *p*-values are found in replication of Edenberg et al’s study [79]. Four of these 14 SNPs have associated genes: *CSMD2* on chromosome 1, *LZTS2* & *PDZD7* on chromosome 10, and *Gcom1* on chromosome 15. There are 34 vs. 11 SNPs that have *p*-values <10*^−^* ^5^. Five SNPs across chromosome 1, 3, 9, 12 and 14 show *p*-values <5.1×10*^−^* ^7^ which is more statistically significant than the case-control analysis by Edenberg et al [79]. Our results also show clusters of SNPs by distance with *p*-values <10*^−^* ^5^ (more than five such markers in one cluster) in genes *EXOC6B*, *FTO*, *NCAM2* and *PPEF1* on chromosome 2, 5, 21 and X, respectively.

**Table 8:** Top SNPs with *p*-values <10*^−^* ^5^ from MH test, and corresponding genes and *p*-values from Case-Control study, dTDT, Unphased association analysis, and IQS with threshold on dosage probabilities above 0.0 or 0.8.

SNP	CHR	Position	Associated Gene	MH test *p*-value	Case-Control *p*-value	dTDT *p*-value	MAF	Unphased Wave I&II	IQS (>0)	IQS (>.8)
rs12116935	1	36,562,133	*FAM176B*	6.98E-06	6.56E-04	1.04E-03	0.38	6.77E-01	0.21	0.91 (395)
rs11583322	1	36,594,899	*STK40*	1.10E-08	7.39E-06	1.04E-04	0.38	8.54E-01	0.20	0.85 (345)
rs1932933	1	160,384,670	*NOS1AP*	5.82E-06	1.99E-04	4.24E-03	0.37	6.85E-01	0.22	0.95 (478)
rs10801629	1	196,110,990		4.13E-06	1.18E-04	9.62E-03	0.40			(482)
rs10922323	1	196,128,944		6.46E-06	1.52E-04	1.23E-02	0.40	9.54E-01	0.31	0.91 (550)
rs1850344	3	108,667,763		8.16E-06	1.33E-04	2.08E-02	0.38	1.13E-01	0.21	0.92 (350)
rs4384980	3	183,941,763		5.78E-07	9.17E-05	6.93E-04	0.42	4.46E-01	0.21	0.90 (405)
rs2857839	4	3,006,428	*GRK4*	6.60E-06	1.11E-03	5.56E-04	0.39			(471)
rs1801058	4	3,008,948	*GRK4*	6.48E-06	9.67E-04	6.57E-04	0.39	5.62E-01	0.22	0.89 (564)
rs2798303	4	3,010,385	*GRK4*	1.28E-06	2.17E-04	8.55E-04	0.42	8.24E-01	0.22	0.91 (615)
rs994029	9	88,565,134		1.98E-07	3.62E-04	1.18E-05	0.37	3.59E-01	0.21	0.89 (713)
rs2398236	10	5,321,159		9.07E-06	7.15E-04	1.96E-03	0.40	5.04E-01	0.22	0.90 (362)
rs9423593	10	5,322,349		8.32E-06	1.04E-03	5.20E-04	0.37	9.04E-01	0.22	0.89 (381)
rs7076488	10	5,323,008		2.13E-06	2.03E-04	1.49E-03	0.41			(562)
rs3781458	10	126,333,921	*FAM53B*	4.27E-06	2.70E-03	1.31E-05	0.37			(729)
rs3781452	10	126,345,119	*FAM53B*	6.86E-06	3.43E-03	1.81E-05	0.37			(449)
rs1503452	11	16,408,708	*SOX6*	3.21E-06	2.58E-04	1.80E-03	0.37	5.38E-02	0.22	0.89 (477)
rs3924047	11	70,507,506	*SHANK2*	8.77E-06	2.19E-04	1.04E-02	0.44	4.95E-01	0.21	0.87 (489)
rs4356270	12	90,843,346		8.42E-06	8.12E-06	1.91E-01	0.35			(370)
rs12427267	12	90,848,103		6.00E-07	6.04E-05	1.90E-03	0.38	2.15E-01	0.19	0.95 (403)
rs11106345	12	90,850,631		7.22E-06	8.83E-06	1.81E-01	0.35			(511)
rs10848190	12	129,767,988		5.43E-06	4.83E-04	2.36E-03	0.39			(327)
rs1035717	14	69,648,452	*SLC8A3*	6.36E-06	1.32E-03	2.19E-04	0.41	7.12E-01	0.23	0.95 (334)
rs4903712	14	77,685,346		1.67E-06	3.40E-05	1.79E-02	0.27	1.99E-03	0.29	0.92 (768)
rs17754467	14	77,692,276		5.54E-07	3.20E-06	4.50E-02	0.23	7.36E-02	0.20	0.87 (621)
rs1568447	17	70,348,607		4.08E-06	5.26E-04	1.13E-03	0.38			(623)
rs9901283	17	70,349,427	*GRIN2C*	9.31E-06	8.18E-04	1.67E-03	0.38			(397)
rs11652088	17	70,351,427	*GRIN2C*	3.50E-06	4.58E-04	1.00E-03	0.38	7.25E-01	0.16	0.82 (537)
rs8111589	19	50,726,398	*OPA3*	8.27E-06	7.50E-05	4.03E-02	0.44	6.08E-01	0.21	0.90 (443)
rs2830045	21	26,380,280	*APP*	6.31E-06	1.77E-03	2.22E-04	0.37			(396)
			**VARIANCE**	9.09E-12	8.60E-07	2.01E-03				
			**MEAN**	5.04E-06	6.94E-04	1.71E-02				

Markers without Unphased and IQS data were not genotyped through the experimental validation. Numbers in the brackets next to the IQS (>0.8) column are the numbers of individuals who meet such restriction on that specific marker.

### Experimental Validation

Based on results from the MH test (5^th^ column on **Table 8**), 19 out of the top 30 SNP markers were genotyped for 1,586 individuals from 220 Wave I & II families. We used the Sequenom MassArray technology for SNP genotyping [81]. PCR primers, extension primers, and multiplexing capabilities were determined with Sequenom MassARRAY Assay Designer software v3.1.2.2. Standard procedures were used to amplify PCR products; unincorporated nucleotides were deactivated with shrimp alkaline phosphatase. A single base pair extension step was completed with the mass extension primer and the terminator (iPLEX). The primer extension products were cleaned with resin and spotted onto a silicon SpectroChip. The chip was scanned with a mass spectrometry workstation (Bruker). The resulting genotype spectra were analyzed with Sequenom SpectroTYPER software v3.4.

Because variant rs11583322 did not work well with the Sequenom genotyping platform, we used the PrimerPicker software [82] to design the assay and followed the protocol described in KASPar SNP Genotyping System manual to run PCR reaction with an ABI GeneAmp PCR System 9700 [83]. Genotypes were accessed using an ABI 7900 HT Fast Real-Time PCR system. Because the genotypes are from linkage families, we used the program UNPHASED [84] to perform a genetic association analysis. Our colleagues in Allison Goate’s lab implemented the above genotyping process. The author did the final analyses of the genotypes. Results are shown in **Table 8**.

## Discussion

### Simulation

As shown above, we can see that simulation can be a powerful tool to investigate many interactions between various factors and help discover potential rules underlying these factors.

With slight modification of the above technique, we can use our simulation to investigate how the dTDT is affected by population stratification. Simulating different populations to have different prevalences, we can choose two sets of trios using different allele frequencies. Applying the dTDT to this combined set of trios, we can test whether the power is lowered or heightened because of the prevalence difference within the populations. Since there is no information on phase of two markers in a trio, we have not introduced the recombination frequency (*θ*) in the simulation.

### Tradeoff

When applying the dTDT to the alcohol dependence data from COGA, nearly twice the number of SNPs (166 vs. 93) were found having *p*-values <10*^−^* ^4^_._ Further, to maximally make use of the available sample data, we combine case-control study and dTDT with the MH test. This potentially increases our sample size and makes the method more robust to uncontrolled factors. As a result, we have one signal in gene STK40 with *p*-value that attains a genome-wide significance level. A large number of SNPs are found having *p*-values <10*^−^* ^4^. Several clusters of SNPs by distance with *p*-values <10*^−^* ^5^ are found in various genes across the genome.

**Figure 7:**
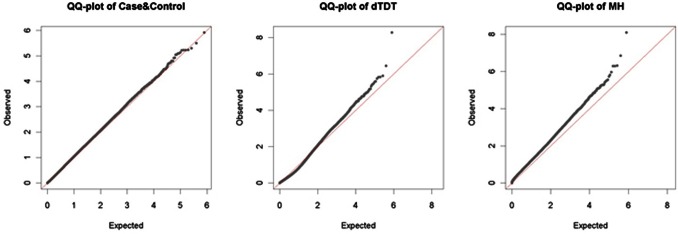
QQ-plots of Case-Control, dTDT and MH tests.

The Quantile-Quantile (Q-Q) plots are used in GWAS to assess the inflation of FPRs by comparing the distribution of observed *p*-values against the theoretical model distribution of expected *p*-values [85]. In theory, without type I error arising from population stratification or other artifacts, the Q-Q plot shall align with the diagonal line. This is true if we use completely randomized data. By comparing the distortion of the Q-Q plot of the test results from this diagonal line, we can tell whether there are false positives or other errors due to genotyping or imputation. Before we draw any conclusion, we provide the Q-Q plots for results from all three Case-Control, dTDT and MH tests on the COGA data (**Figure 7**). From the figure we can see that most of the observed *p*-values from Case-Control and dTDT are along the diagonal line. We do not observe significant distortion, i.e., type I error, in both tests. On the other hand, the Q-Q plot of MH test lies above the diagonal line. As stated earlier, both Case-Control and the dTDT are not robust to the population stratification because of the dependence of allele frequencies in the populations. MH test is basically a combined statistic of these two tests. Though we have increased the sample size in the combined test, we have reason to believe that such sensitivity to population stratification has been inflated in the MH test. This is a tradeoff that we need to pay attention to in the future studies. However, this issue can be partially addressed by restricting accurate genotypes based on the imputation quality score (IQS) [86] (discussed below in *Dealing with errors*).

**Figure 8:**
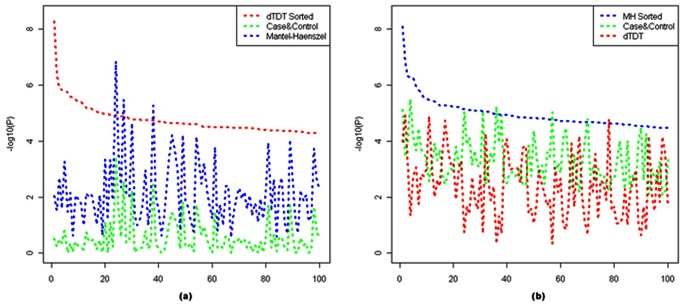
Line chart of top 100 signals from Case-Control, dTDT and MH tests. (a) line chart ranked by -log_10_ (*p-value*) from dTDT and its corresponding -log_10_ (*p-value*) from the other two tests: Case-Control & MH test; (b) line chart ranked by -log_10_ (*p-value*) from MH test and its corresponding -log_10_ (*p-value*) from the other two tests: Case-Control and dTDT.

Having the observed -log_10_ (*p-value*) along the diagonal line in the Q-Q plots doesn’t mean these tests agree with each other. To investigate the concordance among these three tests, we rank the -log_10_ (*p-value*) from the dTDT (or MH test) and pick the top 100 signals. Then we plot these values with their corresponding -log_10_ (*p-value*) from the other two tests (**Figure 8**. To dTDT the other two are Case-Control and MH test; to MH test the other two are Case-Control and dTDT). From the figure, we can see that the concordance performs poorly among these tests. The top signals in either dTDT or MH test do not appear in the top list from the rest two tests as expected. There can be several reasons for this discordance: (1) genotyping errors in both linkage and association data; (2) inaccuracy of the imputation; (3) interference from population stratification. The first two issues may be addressed through experimental validation as discussed below. The last issue requires additional design for the tests that we will discuss in *Conclusions*.

### Dealing with Errors

After correcting for multiple tests (*p*-value = 0.05/19 = 0.0026), SNP rs4903712 on chromosome 14 remained significant. This was the seventh most significant SNP from the MH test. As discussed above, there are several issues affecting the dependability of our test results. As we saw from the Q-Q plots, signals from MH test have been inflated because of double counting of the population stratification factor. On the other hand, the genotyping and imputation accuracy may be taken into account as well. To address these issues, we compute the IQS for the listed top SNP markers on **Table 8** with and without setting a 0.8 threshold on the dosage probabilities. We report the number of individuals who meet such 0.8 threshold (in the last column, the numbers in the brackets next to IQS with dosage probabilities >0.8). According to the IQS, when we exclude the dosage probabilities that are below 0.8, the inference program performs very well and provides above ∼0.90 IQS on average. The reason is that for dosage probabilities that are lower than 0.8, there is too much uncertainty for the program to impute, which not only heavily distorts the results (poor specificity) but also makes it difficult to filter out true positives (low sensitivity). However, there is always a tradeoff when we enhance the accuracy. If we set a 0.8 threshold on dosage probabilities, the sample size dramatically reduces from 1,586 to 326 (intersection set across all markers). We further apply dTDT and MH test onto these 326 individuals with either the inferred or genotyped data but do not find significant markers at a 10*^−^* ^5^ level due to a small sample size (data not shown).

As above, the experimental validation shows that the accuracy of the inference program can heavily impact the results of the dTDT and MH tests. The disagreement of results from these two tests on the real data could be attributed to several sources. First, the sample size of informative data is small. In total, we have 3,567 individuals from 453 EA families included for inference calculation. Within all these individuals, only 471 individuals were selected for SNP validation genotyping. This requires genotypes of more than 85% of individuals to be estimated. In addition, compared to the total number of SNPs, the number of microsatellite markers is also trivial (722 vs. 1 million).

According to **Table 8**, though the sample size may be reduced, we still recommend limiting dosage probabilities before genotyping. In our experience, a threshold at 0.7 ∼ 0.8 level can be a good cut-off. A threshold lower than this level may contribute too much noise and a threshold higher than this may reduce the sample size significantly. Meanwhile, we suggest interpreting the dTDT signals only after genotyping validation in order to lower the risk of false positives.

## Conclusions

Since the discovery of Mendel’s law, genetic research has been challenged to identify genetic variants that contribute to human diseases. Along with the development of genome sequencing technologies, there have been impressive progresses within the research community over the past decade. Numerous methodologies have been developed and many disease-associated genes have been reported [87]. In this study, the work presented here embraces the recent development and addresses some of the research challenges in the field of genetic research. However, as we have seen, despite the promises of the solution we provide, it also prompts a great need to further investigate many of the issues we have presented.

As discussed, traditional case-control studies on GWA often include only unrelated individuals. By including family information in the study, we can expect an increase in power for linkage and association detection. On the other hand, because the traditional TDT requires complete genotypic information from a trio by measuring over-transmission of an allele from heterozygous parents to the affected offspring, it may be less useful in trio data like COGA where there are relatively few complete trios. To overcome these limitations, in this project, we extend the original TDT to the dTDT to accommodate dosage probabilities of a trio. The trio-dosage sets can be inferred through programs like MERLIN. Compared to a recent work from Edenberg et al, the dTDT shows increased power to detect association.

Genotype inference allows us to evaluate the evidence for association at the genetic markers that are not directly-genotyped. It helps improve the power of individual scans and is of particular usefulness for combining information from different studies such as linkage and GWAS. However, the accuracy of genotype inference may be impaired for the following reasons. First, because datasets where subjects are genotyped on different platforms may have different genotyping error rates, when we combine these datasets, inference can be problematic. Second, genotype inference for large datasets based on a small amount of shared information may encounter too much uncertainty in the procedure. For a similar reason, SNPs with low MAF may also have a higher chance of being inferred inaccurately.

On the other hand, a major advantage of the TDT is that it is not susceptible to population stratification. The dTDT is, however, sensitive in that it depends on the marker allele frequencies in the population. Because of this reason, when we combine results from both Case-Control and dTDT, the MH test potentially inflates errors due to population stratification. This can be noticed in the Q-Q plot as we present in **Figure 7**.

In summary, as we inspect the reasons for having low concordance among the tests as well as poor replication from our test results to the experiment validation, we have the following conclusions:

- Because the linkage and GWAS data were genotyped on different platforms, they may carry different genotyping errors, which make it difficult to obtain genotype inference accurately; inference across these platforms can generate spurious associations;
- Due to a great sparsity in the combined dataset, a large number of the markers have to be inferred without sufficient support from the common markers, which introduces too much uncertainty in the inference;
- Because of possible population stratification, combining both the Case-Control and the dTDT to enhance the sample size may introduce false positive signals.

As one solution, when we filter out poorly-inferred SNP markers using IQS, we are able to removes thousands of false positives that can be particularly useful for SNPs with low MAF and when datasets are genotyped on different platforms. However, the tradeoff is we have to exclude a good number of individuals from our database in order to meet such restriction. But this can always be an option when enough samples are available.

Despite this area for methodological development, our work posits that the dTDT has considerable utility for linkage and association testing. By exploiting family data with inference and existing case-control information, the dTDT demonstrated here has opened a new window to possible routes for the integration of both population-based and family-based studies.

### Future Direction

To address the sensitivity issue due to population stratification, it is necessary to validate the SNP genotyping and perform a test such as the PTDT to validate results and use a program such as UNPHASED. This approach minimizes expense when the case/control sample is derived from an existing family study in which relatives have available DNA for typing. Moreover, we may implement additional application to other populations such as African Americans to compare findings with what we have from the European Americans. This will require extending the techniques described above.

We may explore using more of the family data instead of only using SNPs. Other sources of information could be captured, such as the copy number variants (CNVs) [88], [89]. It is also suggested that non-genetic risk factors tend to raise the attention for complex traits and should also be incorporated into the genetic studies. Meanwhile, it is likely that COGA will obtain GWAS data in the relatives so that we can evaluate the efficiency of inference versus having GWAS genotypes available. Power calculation and sample size estimation of the new statistics are needed for general use. Due to the uncertainty inherent to the inference procedure, we plan to develop better strategies for generating dosage probabilities.

Finally, the dTDT should not be limited to dosage probabilities from the inference programs only. As a perspective, the next-generation sequencing data will provide a challenge using the method developed in this paper. Similar methods can be used when pedigree members have a SNP chip, and a subset has sequence data. Despite the discordance and poor replication from our test results, we believe that linkage can help identify regions of interest in conjunction with association testing. Computational inference has helped us reduce the experimental cost in that we may only need to do sequencing on a limited number of family members. Keeping this in mind, we need to implement appropriate selection of the most informative families when we do genotyping. All these future directions shall be promising and have potential to inform the field.

## Supporting Information

Table S1 **Dominant (K  =  0.01).**
(XLSX)

Table S2 **Dominant (K  =  0.1).**
(XLSX)

Table S3 **Dominant (K  =  0.2).**
(XLSX)

Table S4 **Recessive (K  =  0.01).**
(XLSX)

Table S5 **Recessive (K  =  0.1).**
(XLSX)

Table S6 **Recessive (K  =  0.2).**
(XLSX)

Table S7 **Codominant (K  =  0.01).**
(XLSX)

Table S8 **Codominant (K  =  0.1).**
(XLSX)

Table S9 **Codominant (K  =  0.2).**
(XLSX)
